# Whole Genome Sequence-Based Prediction of Resistance Determinants in High-Level Multidrug-Resistant *Campylobacter jejuni* Isolates in Lithuania

**DOI:** 10.3390/microorganisms9010066

**Published:** 2020-12-29

**Authors:** Jurgita Aksomaitiene, Aleksandr Novoslavskij, Egle Kudirkiene, Ausra Gabinaitiene, Mindaugas Malakauskas

**Affiliations:** 1 Department of Food Safety and Quality, Faculty of Veterinary Medicine, Veterinary Academy, Lithuanian University of Health Sciences, Tilzes str. 18, LT 47181 Kaunas, Lithuania; aleksandr.novoslavskij@lsmuni.lt (A.N.); ausra.gabinaitiene@lsmuni.lt (A.G.); mindaugas.malakauskas@lsmuni.lt (M.M.); 2 Department of Veterinary and Animal Sciences, Faculty of Health and Medical Sciences, University of Copenhagen, Stigbøjlen 4, 1870 Frederiksberg C, Denmark; egle@sund.ku.dk

**Keywords:** *Campylobacter jejuni*, multidrug resistance, mobile genetic elements, antibiotic-resistance genes, horizontal gene transfer, genomic islands, whole genome sequence

## Abstract

Spread of antibiotic resistance via mobile genetic elements associates with transfer of genes providing resistance against multiple antibiotics. Use of various comparative genomics analysis techniques enables to find intrinsic and acquired genes associated with phenotypic antimicrobial resistance (AMR) in *Campylobacter jejuni* genome sequences with exceptionally high-level multidrug resistance. In this study, we used whole genome sequences of seven *C. jejuni* to identify isolate-specific genomic features associated with resistance and virulence determinants and their role in multidrug resistance (MDR). All isolates were phenotypically highly resistant to tetracycline, ciprofloxacin, and ceftriaxone (MIC range from 64 to ≥256 µg/mL). Besides, two *C. jejuni* isolates were resistant to gentamicin, and one was resistant to erythromycin. The extensive drug-resistance profiles were confirmed for the two *C. jejuni* isolates assigned to ST-4447 (CC179). The most occurring genetic antimicrobial-resistance determinants were *tetO*, beta-lactamase, and multidrug efflux pumps. In this study, mobile genetic elements (MGEs) were detected in genomic islands carrying genes that confer resistance to MDR, underline their importance for disseminating antibiotic resistance in *C. jejuni*. The genomic approach showed a diverse distribution of virulence markers, including both plasmids and phage sequences that serve as horizontal gene transfer tools. The study findings describe in silico prediction of AMR and virulence genetics determinants combined with phenotypic AMR detection in multidrug-resistant *C. jejuni* isolates from Lithuania.

## 1. Introduction

Campylobacteriosis is one of the most common foodborne bacterial diseases worldwide [1]. The reported confirmed cases of human campylobacteriosis reached 246,571 in 2018 and remained the most frequently reported foodborne illness in the EU with notification rate of 64.1 per 100,000 population. [2]. *Campylobacter jejuni* is an important zoonotic pathogen causing significant infections, especially in young children, geriatric and immunocompromised patients [3,4]. Bacterial antibiotic resistance, especially multidrug resistance (MDR) and extensive drug resistance (XDR), has become a global emerging threat to public health systems [5,6]. Multidrug-resistance bacteria are frequently detected in humans and animals from developed and developing countries and pose a serious threat to human health [7].

Bacteria can acquire antimicrobial resistance through two leading pathways: chromosomal mutation and the acquisition of mobile genetic elements (MGEs) by horizontal gene transfer (HGT). Horizontal gene transfer of mobile genetic elements allows bacteria to exchange the genetic materials among pathogenic and non-pathogenic bacteria from different environments [8,9]. The HGT partly causes an increase in the adaptability of bacteria to environmental changes [10]. The transposition of MGEs can radically alter genome structure and genome sequence of bacteria, as antibiotic resistance is often spread via mobile genetic elements, and carry resistance against multiple antibiotics. Such bacteria with acquired AMR can spread and be transmitted to another environment, as resistant bacteria transfer from wild birds and cattle host to humans [11,12]. A better understanding of antibiotic resistance genes’ (ARGs’) circulation within bacterial species in different host niches and the mobility of these genes between different hosts could be important for identifying and analyzing multidrug resistance [13].

The study aimed to search the possible links among the phenotypic multidrug antimicrobial resistance and whole genome sequencing data (WGS) of *C. jejuni* isolates from different sources (cattle and wild bird feces).

## 2. Materials and Methods

### 2.1. Study Isolates

In total, seven *C. jejuni* isolates from bacterial culture collection of the Department of Food Safety and Quality of Lithuanian University of Health Sciences were tested in this study. These isolates were previously characterized by Multi Locus Sequence Typing (MLST) and assigned to CC179 and CC21 clonal complexes, with wide spread in Lithuania [14,15]. The isolates were stored at −80 °C in brain heart infusion broth (BHI) (Oxoid, Basingstoke, UK) with 30% glycerol (Stanlab, Lublin, Poland). The isolates’ recovery were performed by plating the stocks on Blood agar base No. 2 (Oxoid, Basingstoke, UK) supplemented with 5% defibrinated horse blood (E&O Laboratories Limited, Scotland, UK) and further incubation under microaerophilic conditions (5% oxygen, 10% carbon dioxide, and 85% nitrogen) at 42 °C for 48 h.

### 2.2. Antimicrobial Susceptibility Testing

All isolates were tested for antimicrobial susceptibility to erythromycin, tetracycline, gentamicin, ciprofloxacin, and ceftriaxone (all Sigma-Aldrich, Saint Louis, MO, USA). The minimum inhibitory concentration (MIC) was determined by the agar dilution method performed according to recommendations by the Clinical and Laboratory Standards Institute (CLSI) guidelines [16]. Isolates were cultured on Mueller-Hinton agar (Liofilchem, Italy) plates with dilutions ranging from 0.25 to 256 mg/mL for all antimicrobials. For each individual *C. jejuni* isolate, 5 µL of approximately 1 × 10^7^ CFU/mL (OD600 = 0.1) bacterial suspension dissolved in phosphate-buffered saline (PBS) (E&O Laboratories Limited, Scotland, UK) were spotted onto Mueller-Hinton agar plates containing the corresponding antimicrobial agent concentration. The inoculated plates were incubated under microaerophilic conditions at 42 °C temperature for 24 h. The determination of MIC for each isolate was performed in triplicate. The MIC values were defined as the lowest concentration that produces complete inhibition of *C. jejuni* growth. For quality control, the reference isolate *C. jejuni* ATCC 33,560 was included. These breakpoints defined by the National Antimicrobial Resistance Monitoring System for Enteric Bacteria (NARMS) for antimicrobial susceptibility were used: for erythromycin ≥8 mg/mL, for tetracycline ≥2 mg/mL, for ciprofloxacin ≥1 mg/mL, for gentamicin ≥4 mg/mL, and for ceftriaxone ≥8 mg/mL. Isolates showing resistance to three or more antimicrobials were considered as multidrug-resistant.

### 2.3. Whole Genome Sequencing

DNA extraction was performed using the PureLink Genomic DNA Kit (Invitrogen, Carlsbad, CA, USA) according to the manufacturer’s instructions and finally eluted in 50 µL of sterile Mili-Q water. The concentration and integrity of DNA were quantified by Qubit 3.0 Fluorometer (Thermo Fisher Scientific, Waltham, MA, USA) with the double-stranded DNA (dsDNA) assay HS kit (lifetechnologies, Eugene, OR, USA) and 1% agarose gel, respectively.

According to the manufacturer’s instructions, DNA libraries were prepared using the Nextera XT library preparation kit (Illumina, San Diego, CA, USA). The sequencing was carried out at the NGS-MiSeq core facility of the University of Copenhagen using an Illumina MiSeq platform (Illumina, San Diego, CA, USA) with 250 bp paired-end read format and aiming to obtain an average genome depth of 50X. CLC Genomics Workbench version 6.5.1 was used for the adapter and quality trimming of the raw reads. Sequence reads were de novo assembled into contigs using SPAdes v.3.10 assembler [17]. The quality of the assembly was evaluated with QUAST v.2.3 (3). The subsystems’ annotation was obtained using the SEED-based automated annotation system after the data were uploaded to RAST (Rapid Annotation using Subsystem Technology) [18,19] genome server. Ribosomal multilocus sequence typing (rMLST) employing 53 genes encoding the bacterial ribosome protein subunits (*rps* genes) was performed using the Genome Comparator module of the BIGSdb platform on the PubMLST website [20]. BlastKOALA (https://www.kegg.jp/blastkoala/) [21] was used to perform KO (KEGG Orthology) assignments to characterize individual gene functions and reconstruct KEGG pathways, BRITE hierarchies, and KEGG modules. The presence of potential genes encoding antibiotic resistance was checked using the NCBI AMRFinder v.3.1.1 tool and the ResFinder v.3.0 and PointFinder v.3.1.0 (https://cge.cbs.dtu.dk/services/ResFinder/) [22] databases using thresholds of 90% identity and 60% gene coverage. Besides, a resistome prediction which uses BLAST algorithms to search of the AMR genes and SNPs was performed with Resistance Gene Identifier (RGI v.4.2.2) [23] with a previous coding sequence of the genome submission to the Comprehensive Antimicrobial Resistance Database (https://card.mcmaster.ca/analyze/rgi). Detection of antibiotic-resistance genes, mobile genetic elements, and mutation were performed through alignment to perform multiple alignments of the query with reference sequence downloaded from the NCBI reference (RefSeq) database using the CLUSTAW alignment tool https://www.genome.jp/tools-bin/clustalw [24]. CRISPR finder (https://crispr.i2bc.paris-saclay.fr/Server/) [25] and PathogenFinder 1.1 (https://cge.cbs.dtu.dk/services/PathogenFinder/) [26] databases of the Center for Genomic Epidemiology were used for the potential prediction of pathogenicity. A genome BLAST atlas was generated using isolates of *C. jejuni* comparison against the reference genome NCTC11168 (AL111168.1) using Gview (https://server.gview.ca/) [27]. For the dataset, alignment was used with BLASTn analysis with an e-value of 1 × 10^−10^, coverage 100%, and identity 80%. The IslandViewer version 4 server (https://www.pathogenomics.sfu.ca/islandviewer/) [28] was used to predict the putative genomic islands (GIs).

### 2.4. Data Availability

All genomes were submitted to NCBI under the following Accession Numbers: SAMN08794492; SAMN08794493; SAMN08803042; SAMN08803043; SAMN08803044; SAMN08803060; SAMN08803062 (BioProject PRJNA445645).

## 3. Results and Discussion

### 3.1. Phenotypic Antimicrobial Resistance Determination

All seven *C. jejuni* isolates were resistant to three or more antibiotics, and were identified as multidrug-resistant (MDR). MIC data are provided in Table 1. All isolates were phenotypically highly resistant to tetracycline, ciprofloxacin, and ceftriaxone (MIC range 64 ≥ 256 µg/mL). Besides, two *C. jejuni* isolates were resistant to gentamicin, and one was resistant to erythromycin. The extensive drug-resistance profiles were confirmed for the two *C. jejuni* isolates assigned to ST-4447 (CC179) (Table 2).

### 3.2. Genomics

All isolates with high-level multidrug resistance were further characterized by whole genome sequencing to identify transferable genes encoding antimicrobial resistance.

The general genome stats suggest that genome sizes range from 1.68 to 1.70 Mbp, with the average 30% G + C content, well within the range of available genome sequences. The summary of the genomes’ sequencing assignment is listed in Table 3.

### 3.3. Whole Genome Sequence-Based Genotypic Predictions of Antibiotic-Resistance Genes

**Figure 1:**
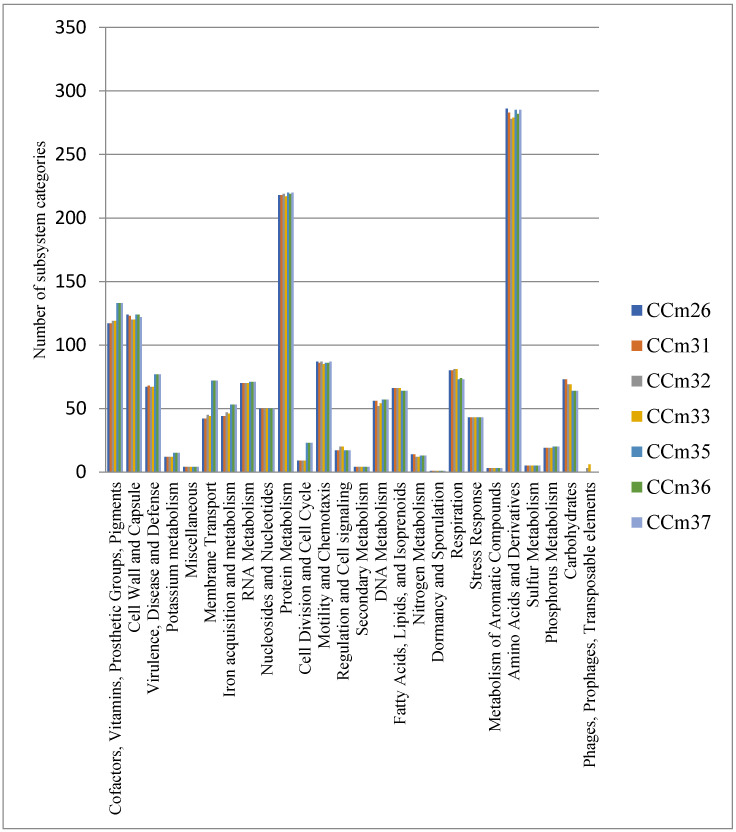
Subsystem category distribution of seven *C. jejuni* isolates.

The genomes of *C. jejuni* isolates were clustered into orthologue groups and annotated in RAST with the aim to identify traits involved in antimicrobial resistance and survival. Based on RAST analysis, 77 genes in three cattle, in each genome assigned to ST-21 (CC21), were annotated in association with virulence, disease, and defense. The analysis revealed the presence of virulence marker genes associated with adhesion (*cadF* and *pEB*1), invasion (*yidC* and *yidD*), and cytotoxin production (*cdtA*, *cdtB*, and *cdtC*). Nine protein-coding genes in genomes of CC_m32_ and CC_m33_ isolates were identified in phages, prophages, and transposable elements’ category including phages’ proteins involved in phage replication process, phage tail, and phage capsid proteins. Figure 1 shows the diagram of the genes associated with the functional categories of examined isolates.

The WGS data of examined isolates were mapped based on intrinsic and acquired genes known to be associated with phenotypic AMR. The genomes were also manually searched for genes known to being involved in AMR and virulence. The isolates CC_m31_ and CC_m32_ assigned to CC179 harbored cobalt-zinc-cadmium resistance determinants composed of *czc*, *chr*, *ncc*, and *mer* genes responsible for resistance to Zn, Cr, Ni and Hg, respectively. In three isolates, CC_m35_, CC_m36_, and CC_m37_, all assigned to CC21 (ST-21), platinum drug resistance *ctpA*, and a cationic antimicrobial peptide (CAMP) system genes cluster were identified (Table 4). The *ctpA* gene encodes the C-terminal processing protease for the photosystem’s D1 protein II reaction center complex related to virulence and cytotoxicity against host cells [29,30].

Type IV secretion system (T4S) genes *virB2*, *virB4*, *virB8* and *virB9* were identified in genomes of *C. jejuni* CC_m33_, CC_m36_, CC_m37_ isolates. The operon of the *cmeABC* multidrug efflux pump, consisting of *cmeA*, *cmeB*, and *cmeC* genes were identified in the genomes of three isolates (Table 4). The *cmeABC*, a resistance-nodulation-division (RND) type of efflux pump, contributes significantly to both intrinsic and acquired resistance to various antimicrobials in *C. jejuni* [31].

Additionally, the *pmrA* efflux pump, which belongs to the resistance-nodulation-division family of transporters and contributes to multidrug resistance of antimicrobials, was found in two *C. jejuni* isolates. The *tetO* gene, which codes the resistance to tetracycline, was detected in all *C. jejuni* isolates from cattle and in one isolate from a wild bird. Another tetracycline resistance gene *tetM* was found in the same two isolates, which had a *tetO* gene. The β-lactamase resistance gene *blaOXA-448* was identified in all *C. jejuni* isolates assigned to clonal complex CC179; however, *blaOXA-61* was identified in all isolates assigned to clonal complex CC21. Among the resistant isolates, several genes coding the virulence factors were found. The chemotaxis and flagellar motility genes, *trg*, *flgE*, and biofilm dispersion *bdlA* gene with increased adherent properties required for biofilm formation, were identified. These genes were identified in all *C. jejuni* isolates, which shows their widespread dissemination throughout *C. jejuni* genomes.

### 3.4. Point Mutation

Nucleotide and amino acid changes of *C. jejuni* genomic sequences are shown in Table 5. All CIP-resistant isolates harbored *gyrA* point mutation T86I. The G→A transversion in the *rpsL* gene was detected in two ST-4447 isolates. The six different point mutations, including deletion Lys→del in the L22 ribosomal protein gene *rpIV*, were observed in ST-6424. The amino acid changes in the *cmeR* gene, including D121N and E159K, were identified in two ST-21 isolates. Fourteen non-synonymous mutations were detected in the *23S rRNA* gene.

### 3.5. Mobile Genetic Elements: Genomic Islands, Prophages and Plasmids

The different genomic approaches analysis revealed the prevalence of MGEs, including plasmids, pathogenicity islands, and bacteriophages in *C. jejuni* isolates. Most of the AMR and virulence factors were distributed within genomic island (GI) regions. Analysis of ARGs’ composition based on GI revealed 18 GIs with the size ranging in length from 5.61 kb to 58.83 kb. The largest GI was detected in the CC_m36_ (58.83 kb), containing gene *tetO* encoding tetracycline resistance, *trg* gene encoding chemotaxis, *virB2* gene, putative DNA-invertase *pinR*, and *ansZ* coding L-asparaginase 2. In total, six GI (size 5.73 to 45.9 kb) were uniquely found in one of the ST-4447 (CC_m33_) isolate, which contains prophage CPS-53 integrase *intS*, *flgE* encoding flagellar motility, *virB2*, *virB4*, *virB8*, and *virB9* genes. GIs play crucial roles in microbial genome evolution and adaptation of microbes to environments as part of a flexible gene pool [32]. Whole genome sequencing also revealed a 131.1 kb phage harbored by one isolate (*C. jejuni* CCm_33_) with high homology (identity 98%; e value 1.37 × 10^−118^) to *Campylobacter* phage PC5 (KX229736.1). Prophages are genomes of temperate phages that have infected a susceptible host bacterium, where they either integrate into the chromosome or exist as circular or linear plasmids [33].

The plasmid of 20,765 bp was identified in two *C. jejuni* isolates assigned to ST-21 (CC21) with high homology of repUS59 (pSSG1) plasmid sequence (FR824044) in the NCBI repository database. Furthermore, the nucleotide sequence comparison of genomic island in CC_m37_ showed presence of pTet plasmids with the *tetO* gene, L-asparginase 2, flagellin A and various homologous hypothetical genes. These acquired elements expand the genetic flexibility of pathogens. Plasmids and bacteriophages contribute to the *C. jejuni* evolution via adaptation and survivability with the novel functions integrated into the chromosome [34,35].

### 3.6. BLAST Identification and Diverse Genomic Locations of the C. jejuni

BLAST genomic atlas provides detailed valuable genomic insights that support genome-wide gene characterization of *C. jejuni* isolates and reveals how similar any genome is to the reference genome (NCTC 11168). Analysis of the G + C content distribution showed localized peak deviations from the genome’s average GC content. At positions from 71,887 to 50,973 bp, the chemotactic transducer gene *pctC* was detected in CC_m33_, CC_m36_, and CC_m37_ isolates. This gene consists of 2102 bp and encodes a transmembrane signaling receptor activity, which is to enhance the ability of signal transduction [36]. Genomic comparison with the reference *C. jejuni* NCTC11168 isolate genome revealed that the *ssa1* gene encoding serotype 1-specific antigen (identity 100%) was found in all ST-21 *C. jejuni* isolates. This serotype-specific antigen has been shown to be involved in the endopeptidase activity regulation complex of *Neisseria meningitides* M0579 and *Pasteurella haemolytica* but is not present in *C. jejuni* NCTC 11168. The multidrug resistance gene *MdtG* (identity 97.88%) was observed in all ST-4447 isolates.

**Figure 2:**
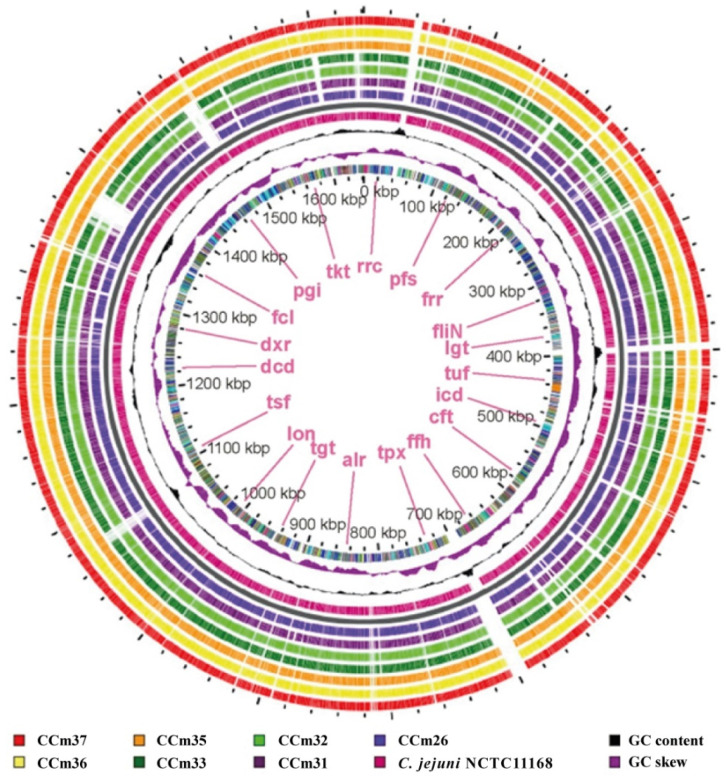
Circular genomic atlas of 7 *C. jejuni* isolates in comparison to the reference *C. jejuni* NCTC11168 genome. The circle is divided into arcs representing the genomes as labeled. The black histogram represents the G + C content, and the purple-green histogram represents the G + C deviation.

Moreover, the BLAST search of *C. jejuni* ST-21 isolates revealed a novel acquired complex that harbors the β-lactamase resistance gene *blaOXA-133* (100% identity) belonging to the class-D β-lactamase family, *yafP* gene, and *ykkC* multidrug resistance gene in the region from 273,300 to 282,500 bases (Figure 2). The changes, most often associated with MGEs that have been acquired by HGT, can initiate a rapid mechanism for acquiring genes and confer novel function [37].

In conclusion, this knowledge provides insights on the distribution and genetic content of MGEs in multidrug *Campylobacter jejuni* isolates. Mobile genetic elements are principally important to facilitate horizontal genetic exchange and, therefore, induce the acquisition and spread of resistance genes. This may allow an assessment of how genes carried by mobile genetic elements can contribute to traits that are responsible for antimicrobial resistance and virulence. These findings highlighted the role of resistance determinants in the epidemiology of MGEs in *C. jejuni* genome sequences and indicate the potential for bacteria’s genomic plasticity. In this context, a better understanding of the acquisition of resistance determinants is critical to understand the capacity of resistance genes to spread in *C. jejuni* population.

## Figures and Tables

**Table 1:** Minimum inhibitory concentration among *Campylobacter jejuni* isolates from cattle and wild birds.

Antimicrobial Agent					
Isolate	TET	CIP	GEN	AXO	ERY
MIC, µg/mL					
CC_m26_	128	128	2	128	4
CC_m31_	>256	64	0.5	128	0.5
CC_m32_	128	256	8	64	8
CC_m33_	>256	128	4	128	0.25
CC_m35_	128	128	0.5	128	0.5
CC_m36_	256	128	0.25	256	0.5
CC_m37_	64	64	0.25	64	0.5
Breakpoint	≥2	≥1	≥4	≥8	≥8

TET, tetracycline; ERY, erythromycin; CIP, ciprofloxacin; GEN, gentamicin; AXO, ceftriaxone; MIC, minimum inhibitory concentration.

**Table 2:** Phenotypic antimicrobial resistance profiles of *C. jejuni* isolates from cattle and wild birds.

Isolate	Source	Clonal Complex	Sequence Type	Antimicrobial Resistance Profile
CC_m26_	Wild bird	CC179	ST-6424	TET + CIP + AXO
CC_m31_	Wild bird	CC179	ST-4447	TET + CIP + AXO
CC_m32_	Wild bird	CC179	ST-4447	TET + CIP + AXO + GEN + ERY *
CC_m33_	Wild bird	CC179	ST-4447	TET + CIP + AXO + GEN *
CC_m35_	Cattle	CC21	ST-21	TET + CIP + AXO
CC_m36_	Cattle	CC21	ST-21	TET + CIP + AXO
CC_m37_	Cattle	CC21	ST-21	TET + CIP + AXO

TET, tetracycline; ERY, erythromycin; CIP, ciprofloxacin; GEN, gentamicin; AXO, ceftriaxone; CC, clonal complex; ST, sequence type; * extensive drug resistant (XRD) isolates.

**Table 3:** List of genomic data of *C. jejuni* isolates.

	CC_m26_	CC_m31_	CC_m32_	CC_m33_	CC_m35_	CC_m36_	CC_m37_
Metrics of sequence data							
Coverage (x)	160	120	180	400	220	180	160
Genomic data report							
Size (bp)	1682833	1685191	1685033	1721979	1701391	1705164	1705114
No. of contigs	31	59	25	29	23	24	21
GC (%)	30.3	30.4	30.4	30.3	30.3	30.3	30.3
N50	122854	62137	158860	184236	116706	122859	135281
L50	4	9	4	4	6	6	5
L75	9	17	8	7	10	9	8
Genes	1768	1772	1770	1814	1781	1781	1779
CDs	1725	1729	1727	1771	1737	1738	1736
Subsystems							
rRNAs	2	2	2	2	2	2	2
tRNAs	40	40	40	40	41	40	40

**Table 4:** Genetic determinants associated of virulence and resistance markers found in *C. jejuni*. *czc*, cobalt zinc cadmium resistance system; *ctpA*, platinum resistance; AMPs, antimicrobial peptides; CAMP, cationic antimicrobial peptide; T4S, Type IV secretion system; *flgE*, flagellar motility; *trg*, chemotaxis; *bdlA*, biofilm formation; +, positive; −, negative; +/−, uncomplete system.

*C. jejuni*	Virulence Markers					Resistance Markers							
	Heavy Metal Resistance		AMPs Sensing System	Invasion		Multidrug Efflux Pupms		Tetracycline		β-Lactams			
	*czc*	*ctpA*	CAMP	T4S	*flgE*, *trg*, *bdlA*	cmeABC	pmrA	*tetO*	*tetM*	*blaOXA-448*	*blaOXA-61*	*blaOXA-451*	*blaOXA-133*
CC_m26_	−	−	−	−	+++	+	+	+	−	+	−	−	−
CC_m31_	+	−	−	−	+++	−	−	−	−	+	−	−	−
CC_m32_	+	−	−	−	+++	−	−	−	−	+	−	−	−
CC_m33_	−	−	−	+/−	+++	−	−	−	−	+	−	−	−
CC_m35_	−	+	+	−	+++	−	−	+	−	−	+	−	+
CC_m36_	−	+	+	+/−	+++	+	−	+	+	−	+	−	+
CC_m37_	−	+	+	+/−	+++	+	+	+	+	−	+	+	+

**Table 5:** Nucleotide and amino acid changes of *C. jejuni* genomic sequences.

L22			*cmeR*			*gyrA*			*rpsL*			*23S rRNA*	
Mutation	Nucleotide Change	Amino Acid Change	Mutation	Nucleotide Change	Amino Acid Change	Mutation	Nucleotide Change	Amino Acid Change	Mutation	Nucleotide Change	Amino Acid Change	Mutation	Nucleotide Change
I165V ^1^	ATT→GTT	Ile→Val	G144D ^1,2,3,4,5^	GGT→GAT	Gly→Asp	R285K ^1,2,3,4^	AGG→AAG	Arg→Lys	A119T ^3,4^	GCT→ACT	Ala→Thr	287G > A ^5,6,7^	G→A
S109A ^1^	TCT→GCT	Ser→Ala	S207G ^1,2,3,4^	AGC→GGC	Ser→Gly	A312T ^1,2,3,4^	GCT→ACT	Ala→Thr				296C > G ^5,6,7^	C→G
T119A ^1^	ACT→GCT	Thr→Ala	D121N ^6,7^	GAC→AAC	Asp→Asn	A664V ^1,2,3,4^	GCC→GTC	Ala→Val				298G > A ^5,6,7^	G→A
T120P ^1^	ACA→CCA	Thr→Pro	E159K ^6,7^	GAA→AAA	Glu→Lys	T665S ^1,2,3,4^	ACT→AGT	Thr→Ser				327G > A ^5,6,7^	G→A
V137A ^1^	GTG→GCG	Val→Ala				T804A ^1,2,3,4^	ACA→GCA	Thr→Ala				364G > C ^5,6,7^	G→C
K123→del ^1^	AAA→del	Lys→del				T86I ^1,2,3,4,5,6,7^	ACA→ATA	Trr→Ile				554A > C ^5,6,7^	A→C
												571T > G ^5,6,7^	T→G
												1027A > G ^5,6,7^	A→G
												1485C > T ^4^	C→T
												1735T > C ^4^	T→C
												1739T > C ^4^	T→C
												1752T > C ^4^	T→C
												1759A > G ^4^	A→G
												1761G > A ^4^	G→A

Superscript numbers indicate the *C. jejuni* isolates harboring specific nucleotide and amino acid changes: ^1^ CCm_26_; ^2^ CC_m31_; ^3^ CC_m32_; ^4^ CC_m33_; ^5^ CC_m35_; ^6^ CC_m36_; ^7^ CC_m37._
